# Re: “Correlations Between Department and Training Program Online Presence and Women in Orthopedic Surgery Training” by Adkins et al.: Social Media As a Tool for Perpetuating Gender Diversity in Orthopedic Residencies

**DOI:** 10.1089/whr.2023.0164

**Published:** 2024-02-15

**Authors:** Cassandra Bakus, Sean Richards, Jasmin Valenti, Nicolas Nadeau, Kevin M. Posner

**Affiliations:** Department of Medical Education, Hackensack Meridian School of Medicine, Nutley, New Jersey, USA.

We want to commend the authors of “Correlations Between Department and Training Program Online Presence and Women in Orthopedic Surgery Training”^1^ for providing valuable insights into what can be done to address the gender disparity within orthopedic residencies. It is well known to be one of the least diverse specialties among race and gender, with only 16% of orthopedic residents being female. In addition, the distribution of women among residencies varies; as of 2018–2019, 12 out of 179 had no women, and 33 programs only had 1.^2,3^

Adkins et al.^1^ discovered a positive correlation between the number of female faculty (MD/DO/PhD) in a program and the number of female residents in the program. Having a proportionally high number of female faculty showcases the program's commitment to diversity and reinforces a welcoming culture of inclusivity, values that women highly regard when applying.^4^ So how can programs display this highly desirable attribute and attract a diverse applicant pool? The answer may reside in one of the phenomena of the 21st century: social media.

The impact of social media is hard to overstate. It is versatile, easy to use, and offers a convenient way to make and spread content. However, this tool may be underused in orthopedic residencies, since in 2020, less than half of programs have a social media account, specifically on Twitter or Instagram.^5^ Owing to the number and geographic spread of orthopedic residency programs, it is unreasonable to expect applicants to visit every program and experience the environment and culture of each themselves. Therefore, social media can provide an intimate view of what life as a female resident looks like.

Programs should focus on posting content that accurately encapsulates all parts of resident life, both inside and outside of the hospital. For example, a day in the life post can depict the everyday roles and responsibilities of residents. Other posts can focus on what life is like outside of the hospital and should include a mix of academic and nonacademic settings, such as journal clubs, conferences, and social events such as mixers and important life events.

Dispersed throughout the content, programs need to ensure that the composition of the faculty is accurately depicted. Programs should not use social media to falsely inflate the diversity of their faculty, but rather use it as a tool to accurately illustrate the environment and existing culture of their program. Social media can give a representation of what life is like for an individual resident and the culture they would contribute to as a member of it.

We wish to provide a simple and actionable task that programs can use to increase the diversity of their incoming applicants and support women in their pursuit of orthopedics. Therefore, programs should take the management of their social media accounts seriously. Programs need to illustrate their commitment to diversity and inclusion, emphasizing their welcoming culture to prospective applicants, and thereby foster growing diversity in the field.
